# Lung and Inferior Vena Cava Point-of-Care Ultrasonography, NT-Pro-BNP, and Discharge Body Weight as Predictors of Rehospitalization in Acute Heart Failure

**DOI:** 10.3390/jcm14144886

**Published:** 2025-07-10

**Authors:** Danilo Martins, Edson Luiz Fávero Junior, Thiago Dias Baumgratz, Cintia Mitsue Pereira Suzuki, Sean Hideo Shirata Lanças, Diego Aparecido Rios Queiroz, Carolina Rorigues Tonon, Taline Lazzarin, Bertha Furlan Polegato, Paula Schmidt Azevedo, Marina Politi Okoshi, Sergio Alberto Rupp de Paiva, Marcos Ferreira Minicucci, Leonardo Antônio Mamede Zornoff

**Affiliations:** Internal Medicine Department, Botucatu Medical School, São Paulo State University (UNESP), Botucatu 18618-970, SP, Brazil; edson.luiz@unesp.br (E.L.F.J.); thiago.d.baumgratz@unesp.br (T.D.B.); cintia.suzuki@unesp.br (C.M.P.S.); sean.lancas@unesp.br (S.H.S.L.); diego.queiroz@unesp.br (D.A.R.Q.); carolina.tonon@unesp.br (C.R.T.); taline.lazzarin@unesp.br (T.L.); bertha.polegato@unesp.br (B.F.P.); schmidt.azevedo@unesp.br (P.S.A.); marina.okoshi@unesp.br (M.P.O.); sergio.paiva@unesp.br (S.A.R.d.P.); marcos.minicucci@unesp.br (M.F.M.); leonardo.zornoff@unesp.br (L.A.M.Z.)

**Keywords:** acute heart failure, point-of-care ultrasound, lung ultrasound, inferior vena cava ultrasound, NT-pro-BNP, body weight, rehospitalization

## Abstract

**Background:** Patients with acute heart failure exhibit high rates of early rehospitalization accompanied by significant mortality. Therefore, identifying high-risk patients who are prone to disease exacerbation may enable early therapeutic interventions for improved disease management. **Methods**: This single-center, prospective observational study was conducted at a Brazilian hospital. Adult patients hospitalized for acute heart failure were enrolled. On the day of hospital discharge, NT-pro-BNP and body weight data were collected, and bedside lung and inferior vena cava ultrasound examinations were performed. The patients were followed up for up to 30 days after discharge. The primary outcome was rehospitalization for acute heart failure. **Results**: A total of 100 patients were included in the final analysis, of whom 10% were readmitted within 30 days owing to acute heart failure. The number of patients with total B-line scores >3 in the readmitted and non-readmitted groups was 6 and 19, respectively (60% and 21%, respectively; absolute risk difference: 39%; *p* = 0.014). The mean inferior vena cava collapsibility index was significantly lower in readmitted compared to that in non-readmitted patients (25.5% vs. 39.8%, standard deviation: 15.4% and 18.4%, respectively; *p* = 0.020). However, mean body weight and mean NT-pro-BNP levels at discharge did not differ between the groups. In a multivariate logistic regression model adjusted for sex, age, discharge body weight, and left ventricular ejection fraction, a total B-lines score >3 had an odds ratio of 4.72 (95% confidence interval (CI): 1.01–22.13; *p* = 0.049), while the inferior vena cava collapsibility index had an odds ratio of 0.96 (95% CI: 0.91–1.01; *p* = 0.091). **Conclusions**: A total B-line score >3 at discharge in patients hospitalized for acute heart failure was associated with 30-day rehospitalization. In contrast, inferior vena cava ultrasound, discharge body weight, and NT-pro-BNP at discharge were not significant predictors of rehospitalization.

## 1. Introduction

Heart failure (HF) is a complex clinical syndrome resulting from structural or functional cardiac abnormalities associated with impaired ventricular ejection or filling capacity [1,2]. It is estimated that over 64 million people have HF worldwide, with a prevalence of 1–3% in the general adult population. Its incidence is estimated to be increasing owing to improved survival rates and longer life expectancy in the general population [3].

Despite advances in HF treatment [4], hospitalization and mortality rates remain high. Approximately 50–75% of patients die within five years. Additionally, approximately 30–40% of patients with HF experience at least one hospitalization episode due to HF [3].

Within the spectrum of HF, acute heart failure (AHF) stands out, characterized by the rapid or gradual onset of HF signs and symptoms that prompt patients to seek urgent care, leading to emergency department visits and unplanned hospital admissions [1,2]. AHF is associated with higher mortality and rehospitalization rates. Approximately 25% of patients hospitalized for AHF die or are readmitted within 30 days of hospitalization [5,6].

Hospital readmissions in these patients were associated with multiple factors. One such factor is failure to identify at-risk patients at discharge [6]. Patients may exhibit subtle signs of HF decompensation on the day of discharge that are not always detected by the attending healthcare team. This failure in identification can result in premature discharge and, consequently, readmissions within a month [6]. Therefore, proper identification of high-risk patients for rehospitalization can assist in optimizing AHF treatment.

Body weight is a key variable potentially associated with a high risk of rehospitalization [7]. Signs of congestion may be reflected in a patient’s excess weight. Both absolute body weight at discharge and its variability during hospitalization may be linked to an increased likelihood of rehospitalization within 30 days [7,8].

Another potential variable that may be associated with hospital readmission in AHF is N-terminal pro-brain natriuretic peptide (NT-pro-BNP) levels [8,9]. N-terminal pro-brain natriuretic peptide is a serum biomarker formed by the cleavage of pro-BNP, a precursor of brain natriuretic peptide primarily produced in the cardiac ventricles. Its levels increase in response to ventricular overload, which is a common phenomenon in patients with HF [10]. Some studies have suggested a correlation between elevated NT-pro-BNP levels and higher hospital readmission rates [6,8].

Recently, bedside ultrasound has become increasingly valuable for non-radiologist and non-cardiologist physicians [11]. It can confirm a diagnosis in more than 50% of suspected cases. Among its various applications, bedside ultrasound is particularly useful for detecting signs of congestion, both pulmonary and systemic, through lung and inferior vena cava (IVC) ultrasound, respectively [12,13].

Lung ultrasonography can detect signs of pulmonary congestion through the presence of B-lines, which are ultrasonographic artifacts arising from increased pulmonary density, a phenomenon commonly observed during pulmonary congestion [13]. Similarly, IVC ultrasound can estimate the IVC collapsibility index, which decreases during ventilation under certain conditions, such as systemic congestion [13]. Thus, both B-lines and the IVC collapsibility index may be associated with higher readmission rates in patients with AHF [14,15].

Although the evidence suggests that discharge body weight, NT-pro-BNP levels, number of B-lines, and the IVC collapsibility index are associated with 30-day rehospitalization and mortality, no studies have investigated the association of all these variables collectively in the same population. The present study aimed to compare the relationship between these variables and 30-day rehospitalization in patients with AHF.

## 2. Material and Methods

This prospective, single-center cohort study was conducted at the Hospital of Botucatu Medical School in Brazil. The study was conducted in accordance with the Declaration of Helsinki and approved by the Research Ethics Committee of Botucatu Medical School (protocol code: 50969621.8.0000.5411, date of approval: 3 November 2021). Data collection commenced following ethical approval.

### 2.1. Inclusion and Exclusion Criteria

All patients hospitalized by the Internal Medicine team for suspected or confirmed decompensated HF were screened for participation in the study. Patients were included if they were over 18 years of age, had a diagnosis of congestive HF, and required intravenous diuretics for the relief of congestive symptoms. Informed consent was obtained from all subjects involved in the study. Congestive HF was defined as the presence of two major criteria or one major and two minor criteria based on the Framingham risk score [16].

The exclusion criteria included a prior diagnosis of cirrhosis, stage V chronic kidney disease, the need for home oxygen therapy, a previous diagnosis of *cor pulmonale*, or an inability to measure body weight on a conventional scale on the day of discharge for any reason. In addition, those with interstitial lung diseases that could generate pulmonary B-lines and interfere with the ultrasonographic analysis of pulmonary congestion, such as COVID-19 or pneumonia, were excluded. Patients who did not undergo bedside ultrasonography on the day of discharge for any reason and those who did not complete the 30-day follow-up period after hospital discharge were also excluded.

### 2.2. Sample Size

For the sample size calculation, an approximate rehospitalization rate of 25% for AHF within 30 days was considered, with a 95% confidence interval (CI) and a sampling error of 10%, using Fisher and van Belle’s formula. The minimum required sample size was calculated to be 73 patients.

### 2.3. Follow-Up Protocol and Outcome

Patients included in the study were followed up at two different time points: at hospital discharge and 30 days after hospital discharge. The 30-day follow-up after discharge was conducted remotely via telephone with the patient or caregiver. The primary outcome assessed was 30-day rehospitalization.

### 2.4. Ultrasound Assessment

Ultrasound assessment was performed on the day the patients were discharged from the hospital using a GE Venue R1 ultrasound machine (GE Healthcare, Chicago, IL, USA). For lung ultrasound, a convex probe (1–5 MHz) was used and the configuration used was the standardized lung mode of the device. The thorax is divided into eight zones, each side having four zones: anterosuperior, anterosuperior, laterosuperior, and lateroinferior [17]. The probe was positioned along the longitudinal plane of each zone. The total B-line score was determined as previously described [18]. If a zone contained fewer than five B-lines, the number of B-lines was counted. If there were five or more B-lines, a default score of 5 was assigned for the zones without completely coalescent B-lines and 10 for the zones with completely coalescent B-lines (white lung signal).

For the IVC ultrasound, the same GE venue R1 model and convex probe were used, but the standard configuration of the device for the abdominal mode was used. The IVC was measured in two-dimensional mode in a subcostal window using the long axis distal to the hepatic vein, approximately 1–3 cm from the entrance to the inferior vena cava in the right atrium [19]. The inspiratory and expiratory diameters, in millimeters (mm), and the collapsibility index, in percentage (%), of the IVC were estimated using the “Auto-IVC” mode of the ultrasound device. The collapsibility index was calculated automatically using the following formula:

CI (%) = [(Dexp − Dinsp)/Dexp] × 100

where CI is the IVC collapsibility index (%), Dexp is the largest IVC diameter during expiration (mm), and Dinsp is the smallest IVC diameter during inspiration (mm). All ultrasound assessments were performed by the same operator, and the images were stored in a file that could only be accessed by the operator. The health assistant team did not have access to the images during the study.

### 2.5. Clinical and Laboratory Variables

Regarding the clinical profile, data were obtained from the medical history, physical examination, and medical records at both admission and discharge. The analyzed variables were age, sex, race, length of hospital stay, body weight, peripheral oxygen saturation, and the need for oxygen therapy for more than 24 h during hospitalization. Body weight measurements were taken using the same portable digital scale for all patients.

The comorbidities investigated included a history of systemic arterial hypertension, diabetes mellitus, dyslipidemia, obesity, past or current smoking, and past or current alcohol consumption. Patients were classified as diabetic if they had fasting glucose levels ≥126 mg/dL or casual glucose levels ≥200 mg/dL in previous or hospitalization tests, or if they had a prior diagnosis and were undergoing treatment. Hypertension was defined as systolic blood pressure ≥140 mmHg and/or diastolic blood pressure ≥90 mmHg, or a prior diagnosis of hypertension. Obesity was defined as a body mass index ≥30 kg/m^2^, and dyslipidemia was considered in patients with a prior diagnosis of dyslipidemia and/or those undergoing LDL cholesterol-lowering therapies.

The heart failure parameters investigated included ejection fraction (%) and etiology, as determined by echocardiography. Ejection fraction was assessed using the most recent echocardiogram performed before hospitalization from the time of enrollment of the patient in the study. Patients were classified as having hypertensive HF, ischemic HF, valvular HF, dilated cardiomyopathy, or other etiologies, which were determined based on clinical history and echocardiographic findings.

Medication use was also analyzed, including angiotensin-converting enzyme inhibitors, angiotensin receptor blockers, neprilysin inhibitors combined with valsartan, beta-blockers, calcium channel blockers, nitrates, vasodilators, positive inotropes, diuretics, antiplatelet agents, statins, insulin, sodium; glucose cotransporter 2 inhibitors (SGLT2Is), and other oral antidiabetic drugs.

Laboratory parameters conducted at hospital admission that were analyzed included hemoglobin (g/dL), hematocrit (%), platelet count (/mm^3^), leukocyte count (/mm^3^), serum creatinine (mg/dL), serum urea (mg/dL), serum sodium (mmol/L), serum potassium (mmol/L), and serum NT-pro-BNP (pg/mL). The laboratory variables at hospital discharge that were also analyzed included serum creatinine (mg/dL), serum urea (mg/dL), serum sodium (mmol/L), serum potassium (mmol/L), and serum NT-pro-BNP (pg/mL).

### 2.6. Statistical Analysis

Enrolled patients were categorized into two groups: those who were rehospitalized within 30 days and those who were not. Proportional variables were analyzed using either the χ^2^ test or Fisher’s exact test. Continuous variables were tested for normality using the Shapiro–Wilk test. Variables with a normal distribution were expressed as means ± standard deviations, and group comparisons were performed using a Student’s *t*-test. For non-parametric variables, medians and interquartile ranges were calculated, and group comparisons were conducted using the Mann–Whitney U test. Associations between clinical and ultrasonographic variables and clinical outcomes were analyzed using univariate and multivariate logistic regression. Variables that showed statistically significant differences on univariate analysis were initially included in the multivariate analysis. In the second model, the independent variables recognized as clinically relevant in the context of AHF were sex, age, body weight at discharge, and left ventricular ejection fraction. All statistical analyses were performed using SigmaPlot for Windows v12.0, with a significance level of 5% adopted for all tests.

## 3. Results

**Figure 1:**
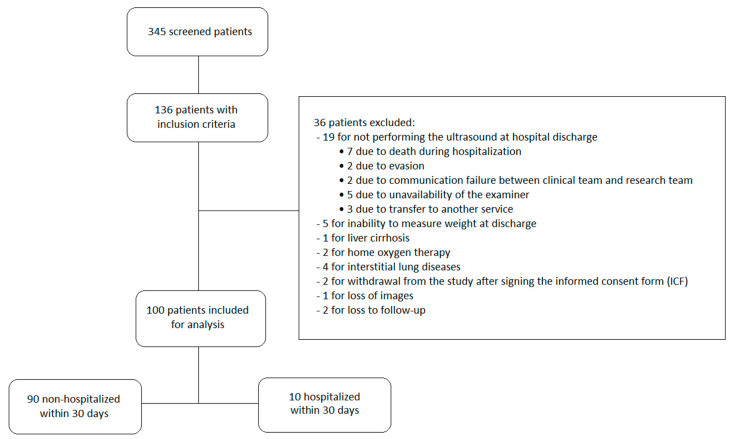
Flowchart of patient inclusion and exclusion in the study.

Between 6 January 2022, and 3 January 2024, 345 patients were screened. Of them, 136 met the inclusion criteria. However, thirty-six patients were excluded: five due to the inability to measure body weight at discharge, one due to hepatic cirrhosis, two due to the need for home oxygen therapy after discharge, four due to concomitant interstitial lung disease that could interfere with pulmonary congestion analysis, nineteen due to not undergoing ultrasound at discharge, two due to withdrawal of informed consent after signing, one due to the loss of recorded ultrasound images caused by technical issues with the ultrasound device, and two due to loss of follow-up. As a result, 100 patients were included in the final data analysis (Figure 1).

### 3.1. Clinical and Laboratory Characteristics

Among the 100 patients included in the analysis, the median age was 70 years (interquartile range: 62–79 years); 58% were female, and 78% were Caucasian. Within 30 days post discharge, 10% of patients were rehospitalized, with all cases attributed to HF decompensation. Table 1 presents the clinical characteristics, outcomes, and differences between rehospitalized and non-rehospitalized patients. No statistically significant clinical differences were observed between the groups.

Regarding laboratory differences, only serum potassium at discharge showed a statistically significant difference, with a mean value of 4.6 (±0.7) mmol/L for rehospitalized patients and 4.2 (±0.6) mmol/L for non-rehospitalized patients (*p* = 0.046). Table 2 summarizes the laboratory characteristics of the participants and differences between the rehospitalized and non-rehospitalized groups.

### 3.2. Ultrasound Characteristics

There was a significant difference in the total B-line scores between the two groups at discharge. The rehospitalized group had a median score of 5 (interquartile range: 0–11), compared with 1 (interquartile range: 0–3) for the non-rehospitalized group (*p* = 0.042). Among the rehospitalized patients, six (60%) presented with total B-line scores >3, compared to nineteen patients (21%) in the non-rehospitalized group (*p* = 0.014).

In the IVC assessment, four patients were unable to undergo the examination due to technical difficulties (all from the non-rehospitalized group). Statistically significant differences were observed between the groups regarding the IVC collapsibility index, proportion of patients with an IVC collapsibility index >30%, and inspiratory diameter of the IVC. The mean IVC collapsibility index was 25.5 ± 15.4% for the rehospitalized group and 39.8 ± 18.4% for the non-rehospitalized group (*p* = 0.020). The number of patients with an IVC collapsibility index >30% was three (30%) in the rehospitalized group and fifty-eight (67%) in the non-rehospitalized group (*p* = 0.034). Finally, the median inspiratory diameter of the IVC was 18.4 mm (interquartile range: 5.3–20.6) in the rehospitalized group and 8.1 mm (interquartile range: 5.5–13.7) in the non-rehospitalized group (*p* = 0.048). Table 3 summarizes the thoracic and IVC ultrasound characteristics of the patients and the differences between the rehospitalized and non-rehospitalized groups.

### 3.3. Discharge Body Weight and NT-Pro-BNP Level

The rehospitalized group had a median body weight at discharge of 74.0 kg (interquartile range: 63.2–84.1 kg) and a median NT-pro-BNP serum level of 5.515 pg/mL (interquartile range: 2.067–9.490 pg/mL). In contrast, the non-rehospitalized group presented a median body weight at discharge of 71.4 kg (interquartile range: 61.7–83.9 kg) and a median NT-pro-BNP serum level of 2.550 pg/mL (interquartile range: 900–4.225 pg/mL). Neither discharge body weight nor serum NT-pro-BNP levels showed a statistically significant difference between the groups (*p* = 0.850 and *p* = 0.079, respectively).

Regarding the variability in body weight and NT-pro-BNP, 87% of the included patients had their weight measured during the study triage, and 96% had their NT-pro-BNP levels assessed upon hospital admission. Although both groups demonstrated body weight loss and a reduction in NT-pro-BNP levels during hospitalization, the variability in these parameters did not differ significantly between the groups. Variability in body weight (Table 1) and NT-pro-BNP levels (Table 2) yielded *p*-values of 0.368 and 0.815, respectively.

### 3.4. Logistic Regression Model

Multivariate logistic regression was performed to identify predictors of rehospitalization within 30 days in patients admitted with AHF (Table 4). According to the analysis, the number of B-lines in the lungs did not show a statistically significant difference between groups when adjusted for serum potassium concentration at discharge (odds ratio—OR: 1.08; 95% confidence interval—95% CI: 0.97–1.20; *p* = 0.159) or when adjusted for age, sex, left ventricular ejection fraction, and discharge body weight (OR: 1.08; 95% CI: 0.97–1.21; *p* = 0.174). However, when considering patients with >3 B-lines, a statistically significant difference in rehospitalization within 30 days was found both after adjusting for serum potassium at discharge (OR: 5.15; 95% CI: 1.27–20.85; *p* = 0.022) and after adjusting for age, sex, left ventricular ejection fraction, and discharge body weight (OR: 4.72; 95% CI: 1.01–22.13; *p* = 0.049).

Regarding IVC ultrasound, both the collapsibility index (OR: 0.95; 95% CI: 0.91–0.99; *p* = 0.041) and the category of patients with a collapsibility index >30% (OR: 0.19; 95% CI: 0.04–0.84; *p* = 0.029) maintained statistically significant differences after adjusting for serum potassium at discharge. However, when adjusted for age, sex, left ventricular ejection fraction, and discharge body weight, neither the collapsibility index (OR: 0.96; 95% CI: 0.91–1.01; *p* = 0.091) nor the category of collapsibility index >30% (OR: 0.29; 95% CI: 0.06–1.29; *p* = 0.104) showed statistically significant differences for predicting rehospitalization within 30 days.

## 4. Discussion

The main objective of this study was to evaluate the association of thoracic and IVC ultrasound findings, body weight at discharge, and serum NT-pro-BNP levels at discharge with the 30-day rehospitalization rate in patients admitted for AHF. Among these variables, only the ultrasonographic measures (total B-line score and IVC collapsibility index) were associated with higher rehospitalization rates within 30 days during univariate analysis. However, only pulmonary ultrasound, using the categorization of groups with total B-line scores >3, was associated with higher rehospitalization rates, after adjusting for confounding factors. Although other studies have assessed the impact of B-lines at hospital discharge, this was the first study to use a simpler technique (the eight-zone method) and compare it to a 30-day outcome.

### 4.1. Lung Ultrasound

Although the number of B-lines in the lungs was not associated with higher 30-day rehospitalization rates after adjustment, patients with higher total B-line scores had higher rehospitalization rates within 30 days. This difference is likely because not all B-lines are pathological. B-lines are ultrasonographic artifacts that form because of thickening of the subpleural interlobular septum [20]. The presence of up to two B-lines in each lung is considered normal because this finding can represent the visualization of interlobar fissures [13,21]. When more than three B-lines are present, the examination is considered abnormal, indicating a pathological process leading to the thickening of the interlobular septum. The primary cause of this thickening is the presence of extravascular pulmonary fluid, which is mostly caused by cardiogenic pulmonary edema. However, it is important to note that other conditions, such as acute respiratory distress syndrome, can also lead to the formation of B-lines [13,20,21]. To minimize confounding ultrasonographic images, we excluded clinical conditions that could interfere with the formation of B-lines from non-heart failure etiologies, such as interstitial lung diseases and cirrhosis.

To date, few studies have evaluated the role of pulmonary ultrasound in predicting rehospitalization due to AHF. A recent systematic review included nine studies to assess the prognosis of pulmonary ultrasound in patients with heart failure [15], but only three of them evaluated the outcomes of rehospitalization and mortality in patients with AHF. The meta-analysis concluded that the presence of B-lines at discharge is a good predictor of rehospitalization and mortality in patients with AHF, a result similar to that found in the present study. However, the studies evaluated used the 28-zone assessment method, which, although considered more accurate, is a more labor-intensive method for lung evaluation than the eight-zone method. The present study opted for the eight-zone method due to its practicality and achieved similar results.

Recently, two randomized clinical trials evaluated AHF treatment guided by pulmonary ultrasound, with uncertain results [22,23]. The multicenter BLUSHED AHF study compared pulmonary ultrasound-guided treatment for AHF with standard clinical treatment. Although the proposed protocol showed earlier resolution of congestion, there was no difference between the groups in terms of days free from hospitalization at 30 days of follow-up [22]. In contrast, the unicentric CAVAL US-AHF study evaluated AHF treatment guided by both chest and inferior vena cava ultrasound and showed a significant reduction in the combined outcome, including readmission, unplanned visits to emergency departments for AHF decompensation, and death within 90 days [23]. Despite its positive findings, the study evaluated the combined outcome over a longer follow-up period (90 days). Our study contributes to the earlier analyses and focuses on a single outcome.

### 4.2. Inferior Vena Cava Ultrasound

The first study to evaluate the prognosis of patients hospitalized for AHF using IVC ultrasound, published in 2008, was a prospective observational study that showed an association between a lower IVC collapsibility index and higher rehospitalization rates [24]. The study also showed a significant association between the IVC diameter and 30-day readmission rate for AHF. Since then, few studies have been published on this topic, and no systematic reviews have been conducted. Among these studies, the only randomized clinical trial that included a treatment protocol with IVC assessment was the CAVAL US-AHF study [23]. However, it is important to highlight that the IVC was not assessed in isolation. Instead, participants were classified based on both pulmonary and IVC findings, making it impossible to assert that IVC ultrasonography is a good predictor of rehospitalization. Furthermore, observational studies have reported contradictory results. For example, Patnaik et al. evaluated 106 patients hospitalized for AHF and found no statistically significant difference in predicting 30-day rehospitalization rates when using a 50% IVC collapsibility index cutoff [25].

The present study showed an association between the IVC collapsibility index and 30-day rehospitalization only in the univariate analysis. This association remained significant after adjusting for serum potassium levels at hospital discharge, the only laboratory variable that showed a statistically significant difference between the rehospitalized and non-rehospitalized groups. However, after adjusting for sex, age, discharge body weight, and left ventricular ejection fraction, no association was found between the IVC collapsibility index and rehospitalization outcomes. One possible explanation for this lack of association in the multivariate model could be that IVC collapsibility is influenced by factors other than congestion, such as intra-abdominal pressure variations among patients [19]. Therefore, further studies are required to better assess the role of IVC ultrasound in predicting rehospitalization risk in patients with AHF.

### 4.3. Discharge Body Weight and NT-Pro-BNP Level

Body weight is a key variable in managing patients hospitalized for AHF, both for the treatment of acute conditions and determining prognosis. It is recommended that, during hospitalization, the patient’s body weight be monitored and that the patient lose about 0.5 to 1.0 kg per day in response to diuretic treatment until reaching their dry weight [2]. Despite this recommendation, few studies have assessed the prognostic value of body weight at discharge. In an observational study, Naderi et al. concluded that among the various risk factors for rehospitalization within 6 months in patients hospitalized for AHF, absolute body weight at discharge was not a good predictor after adjusting for confounding variables, a result consistent with our study results [8]. One possible explanation is that absolute body weight at discharge may not necessarily reflect the patient’s dry weight.

When considering body weight as a prognostic marker, a potentially better variable is the variation in body weight during hospitalization. Gill et al. conducted a retrospective study evaluating more than 8000 patients hospitalized for AHF and categorized them into two groups based on body weight loss during hospitalization. The group that experienced a loss of more than 1.0 kg during hospitalization had lower rehospitalization rates at 6 months [7]. However, this was not observed in the present study, possibly because of several limitations. The first was the difficulty in measuring body weight at patient admission. Patients often face significant limitations in body weight measurement owing to their cardiovascular conditions, such as rest dyspnea. Additionally, it is common for patients with heart failure to experience frailty associated with their cardiovascular condition [26] as well as other physical limitations, which prevent them from being weighed on conventional scales. In the present study, 13% of the analyzed patients did not have their body weights measured during screening, which made prognostic evaluation of this variable unfeasible. The second limitation is the difficulty in determining whether a patient has truly reached their dry weight on the day of hospital discharge. Although patients may experience significant weight loss during hospitalization, they may still have subtle signs of congestion that were not identified during the discharge clinical evaluation. In such cases, the use of more accurate instruments such as pulmonary ultrasound to detect pulmonary congestion may be better for identifying patients who are still congested [27]. This hypothesis is supported by the results of our study, in which the presence of B-lines was associated with rehospitalization at 30 days, in contrast to body weight at discharge and body weight variation during hospitalization. To date, this study is the first to perform a comparative analysis of the presence of B-lines with patient body weights throughout hospitalization.

Another variable that has been increasingly studied is the serum NT-pro-BNP level. Thus, NT-pro-BNP is a good test for establishing a diagnosis of HF, particularly in doubtful cases [1]. However, its role as a prognostic marker in hospitalized patients with acute HF remains unclear. Although there is evidence pointing to an association between serum NT-pro-BNP levels at hospital discharge and rehospitalization and post-discharge mortality rates [28], it is still not possible to determine the optimal cutoff value for defining prognosis [29]. A recent systematic review showed that despite this association, the studies included had a high risk of bias [29]. In the present study, neither the absolute NT-pro-BNP value at discharge nor its variation during hospitalization were associated with rehospitalization, suggesting that even if the test has some predictive value, it is likely not superior to bedside ultrasound.

### 4.4. Other Risk Factors

One of the risk factors identified in the present study that showed an association in the univariate analysis between the rehospitalized and non-rehospitalized groups was the serum potassium level at hospital discharge. This finding may represent a sign of worse prognosis in patients with AHF and could indicate indirect congestion, as serum potassium levels tend to increase in patients with reduced diuresis [30]. However, in the present study, the difference in serum potassium levels between the groups, although statistically significant, was clinically irrelevant, with an absolute mean difference of 0.4 mmol/L. Furthermore, this difference, when used to adjust for correlations, did not alter the results of the logistic regression for the ultrasonographic variables.

### 4.5. Study Limitations

The present study had some limitations. First, it was a single-center study, which could limit the generalizability of the findings to other populations. Second, the sample size was relatively small, although a statistically significant association was still found for pulmonary ultrasonography results. Finally, some patients had missing data, such as body weight measurement at admission and evaluation of the collapsibility index at discharge, which could have influenced the analysis. However, despite these limitations, we believe that our study makes important contributions to the field, suggesting that pulmonary ultrasonography is superior to inferior vena cava ultrasonography in terms of body weight and NT-pro-BNP levels as predictors of 30-day rehospitalization after hospitalization for AHF.

## 5. Conclusions

A total B-line score >3, assessed using bedside pulmonary ultrasonography at hospital discharge in patients hospitalized for AHF, was associated with a higher 30-day rehospitalization rate. In contrast, the collapsibility indexes measured using bedside IVC ultrasonography, NT-pro-BNP levels, and body weight at discharge in patients hospitalized for AHF were not associated with 30-day rehospitalization. These results emphasize the importance of including ultrasonography in the management of hospitalized patients with acute heart failure, especially lung ultrasonography. Also, it may assist physicians to take better decisions on discharging heart failure patients.

## Figures and Tables

**Table 1:** Clinical characteristics of patients, explored outcomes, and differences between rehospitalized and non-rehospitalized groups.

	Total (*n* = 100)	Non-Rehospitalized (*n* = 90)	Rehospitalized (*n* = 10)	*p*-Value
Age, years	70 (62–79)	70 (64–79)	65 (50–72)	0.151
Woman, *n*	58 (58)	55 (61)	3 (30)	0.090
Caucasian, *n*	78 (78)	70 (78)	8 (80)	1.000
Length of stay, days	7 (5–11)	7 (4–11)	10 (7–16)	0.062
Use of oxygen during hospitalization, *n*	53 (53)	49 (54)	4 (40)	0.509
Body weight at screening, kg	72.1 (62.2–86.3) *	71.3 (60.8–87.2) **	76.1 (63.7–83.5)	0.576
Body weight at discharge, kg	72.0 (61.9–83.4)	71.4 (61.7–83.9)	74.0 (63.2–84.1)	0.850
Variation in body weight from screening to discharge, kg	−0.4 (−2.0–+0.1) *	−0.4 (−1.9–+0.1) **	−0.8 (−3.2–−0.2)	0.368
Peripheral oxygen saturation at discharge, %	95 (93–96)	95 (93–96)	95 (94–97)	0.179
**Heart failure**				
Eject fraction, %	46 (30–64)	52 (30–65)	33 (26–56)	0.132
Ischemic, *n*	33 (33)	28 (31)	5 (50)	0.291
Hypertensive, *n*	34 (34)	31 (34)	3 (30)	1.000
Valvular, *n*	7 (7)	7 (8)	0 (0)	1.000
Dilated cardiomyopathy, *n*	13 (13)	11 (12)	2 (20)	0.615
Others, *n*	14 (14)	13 (14)	1 (10)	1.000
**Comorbidities, *n***				
Hypertension	17 (17)	15 (17)	2 (20)	0.677
Diabetes Mellitus	53 (53)	47 (52)	4 (40)	0.521
Dyslipidemia	28 (28)	24 (27)	4 (40)	0.460
Obesity	36 (36)	32 (36)	4 (40)	0.744
Current or previous smoking	55 (55)	50 (56)	5 (50)	0.750
Current or previous alcoholism	20 (20)	17 (19)	3 (30)	0.414
**Medications to be used prior to hospitalization, *n***				
ACEI	25 (25)	24 (27)	1 (10)	0.444
ARB	36 (36)	32 (36)	4 (40)	0.744
Beta-blockers	51 (51)	47 (52)	4 (40)	0.521
Aspirin	34 (34)	31 (34)	3 (30)	1.000
Statin	47 (47)	43 (48)	4 (40)	0.746
Aldosterone antagonist	19 (19)	18 (20)	1 (10)	0.682
Calcium channel blockers	20 (20)	17 (19)	3 (30)	0.414
Diuretics	66 (66)	59 (66)	7 (70)	1.000
Neprilysin inhibitors combined with valsartan	0 (0)	0 (0)	0 (0)	-
Nitrate	7 (7)	7 (8)	0 (0)	1.000
Vasodilator	6 (6)	5 (6)	1 (10)	0.478
SGLT2 Inhibitor	11 (11)	11 (12)	0 (0)	0.596
Other oral antidiabetics	42 (42)	38 (42)	4 (40)	1.000
Insulin	29 (29)	26 (29)	3 (30)	1.000

ARB: angiotensin II receptor blocker; ACEI: angiotensin-converting enzyme inhibitor; * Data refers to a total of 87 patients. ** Data refers to a total of 77 patients.

**Table 2:** Laboratory characteristics and differences between rehospitalized and non-rehospitalized groups.

	Total (*n* = 100)	Non-Rehospitalized (*n* = 90)	Rehospitalized (*n* = 10)	*p*-Value
**Admission laboratories**				
Hemoglobin, g/dL	12.9 (±2.2)	13.0 (±2.2)	12.3 (±2.3)	0.389
Hematocrit, g/dL	39.4 (±6.3)	39.6 (±6.1)	38.0 (±7.1)	0.473
Platelets, /mm^3^	227.500 (191.000–261.750)	228.500 (193.000–261.250)	214.500 (175.250–268.250)	0.577
Leucocytes, /mm^3^	9.250 (7.200–11.950)	9.200 (7.275–11.850)	9.950 (6.625–18.950)	0.705
Creatinine, mg/dL	1.1 (0.8–1.6)	1.1 (0.8–1.5)	1.1 (0.9–3.5)	0.300
Urea, mg/dL	58 (35–77)	59 (35–77)	47 (38–124)	0.679
Sodium, mmol/L	138 (135–140)	138 (136–140)	136 (131–140)	0.416
Potassium, mmol/L	4.3 (3.8–4.8)	4.3 (3.8–4.7)	4.8 (4.1–5.7)	0.088
NT-pro-BNP, pg/mL	5.995 (3.355–11.275) *	5.770 (3.257–11.225) **	9.505 (3.420–13.500)	0.408
**Discharge laboratories**				
Creatinine, mg/dL	1.1 (0.8–1.5)	1.1 (0.8–1.4)	1.1 (0.8–2.8)	0.447
Urea, mg/dL	54 (38–77)	53 (38–75)	58 (36–95)	0.633
Sodium, mmol/L	136 (134–138)	136 (134–138)	135 (132–136)	0.076
Potassium, mmol/L	4.3 (±0.6)	4.2 (±0.6)	4.6 (±0.7)	**0.046**
NT-pro-BNP, pg/mL	2.620 (970–4.587)	2.550 (900–4.225)	5.515 (2.067–9.490)	0.079
Variation in NT-pro-BNP from admission to discharge, pg/mL	−3.063 (−7.062–−746) *	−3.063 (−7.137–−729) **	−2.510 (−6.077– −1.082)	0.815

NT-pro-BNP: aminoterminal-pro-brain natriuretic peptide. * Data refers to a total of 96 patients. ** Data refers to a total of 86 patients. Bold values refer to variables with statistical significance (*p* < 0.05).

**Table 3:** Characteristics of chest ultrasound and IVC ultrasound and differences between the rehospitalized and non-rehospitalized groups.

	Total	Non-Rehospitalized	Rehospitalized	*p*-Value
**Lung ultrasound**	***n* = 100**	***n* = 90**	***n* = 10**	
Total B-lines score, *n*	1 (0–4)	1 (0–3)	5 (0–11)	**0.042**
Presence of B-lines, *n*	57 (57)	50 (56)	7 (70)	0.508
Total B-lines score > 3, *n*	25 (25)	19 (21)	6 (60)	**0.014**
**IVC ultrasound**	***n* = 96**	***n* = 86**	***n* = 10**	
Collapsibility index, %	38.3 (±18.6)	39.8 (±18.4)	25.5 (±15.4)	**0.020**
Collapsibility index > 30%, *n*	61 (64)	58 (67)	3 (30)	**0.034**
Inspiratory diameter, mm	9.3 (5.5–14.6)	8.1 (5.5–13.7)	18.4 (5.3–20.6)	**0.048**
Expiratory diameter, mm	16.3 (11.1–20.0)	15.9 (11.2–19.1)	21.3 (9.2–24.9)	0.135

IVC: inferior vena cava. Bold values refer to variables with statistical significance (*p* < 0.05).

**Table 4:** Logistic regression model for predicting 30-day rehospitalization in patients hospitalized for acute heart failure.

Variable	OR	95% CI	*p*-Value
**Lung ultrasound (*n* = 100)**			
Total B-lines score ^1^	1.11	1.01–1.22	**0.048**
Total B-lines score ^2^	1.08	0.97–1.20	0.159
Total B-lines score ^3^	1.08	0.97–1.21	0.174
Total B-lines score > 3 ^1^	5.60	1.43–21.90	**0.013**
Total B-lines score > 3 ^2^	5.15	1.27–20.85	**0.022**
Total B-lines score > 3 ^3^	4.72	1.01–22.13	**0.049**
**IVC ultrasound (*n* = 96)**			
IVC collapsibility index ^1^	0.95	0.90–0.99	**0.029**
IVC collapsibility index ^2^	0.95	0.91–0.99	**0.041**
IVC collapsibility index ^3^	0.96	0.91–1.01	0.091
IVC collapsibility index > 30% ^1^	0.21	0.05–0.86	**0.030**
IVC collapsibility index > 30% ^2^	0.19	0.04–0.84	**0.029**
IVC collapsibility index > 30% ^3^	0.29	0.06–1.29	0.104

IVC: inferior vena cava; CI: confidence interval. ^1^ Unadjusted analysis. ^2^ Analysis adjusted for serum potassium at hospital discharge. ^3^ Analysis adjusted for age, sex, left ventricular ejection fraction, and weight at discharge. Bold values refer to variables with statistical significance (*p* < 0.05).

## Data Availability

The datasets used and analyzed during the current study are available from the corresponding author on reasonable request.

## References

[1] McDonagh T.A., Metra M., Adamo M., Gardner R.S., Baumbach A., Böhm M., Burri H., Butler J., Čelutkienė J., Chioncel O. (2021). 2021 ESC Guidelines for the diagnosis and treatment of acute and chronic heart failure. Eur. Heart J..

[2] Heidenreich P.A., Bozkurt B., Aguilar D., Allen L.A., Byun J.J., Colvin M.M., Deswal A., Drazner M.H., Dunlay S.M., Evers L.R. (2022). 2022 AHA/ACC/HFSA Guideline for the Management of Heart Failure: Executive Summary: A Report of the American College of Cardiology/American Heart Association Joint Committee on Clinical Practice Guidelines. Circulation.

[3] Savarese G., Becher P.M., Lund L.H., Seferovic P., Rosano G.M.C., Coats A.J.S. (2023). Global burden of heart failure: A comprehensive and updated review of epidemiology. Cardiovasc. Res..

[4] McDonagh T.A., Metra M., Adamo M., Gardner R.S., Baumbach A., Böhm M., Burri H., Butler J., Čelutkienė J., Chioncel O. (2023). 2023 Focused Update of the 2021 ESC Guidelines for the diagnosis and treatment of acute and chronic heart failure. Eur. Heart J..

[5] Greene S.J., Bauersachs J., Brugts J.J., Ezekowitz J.A., Lam C.S.P., Lund L.H., Ponikowski P., Voors A.A., Zannad F., Zieroth S. (2023). Worsening Heart Failure: Nomenclature, Epidemiology, and Future Directions. J. Am. Coll. Cardiol..

[6] Dunbar-Yaffe R., Stitt A., Lee J.J., Mohamed S., Lee D.S. (2015). Assessing Risk and Preventing 30-Day Readmissions in Decompensated Heart Failure: Opportunity to Intervene?. Curr. Heart Fail. Rep..

[7] Gill G.S., Lam P.H., Brar V., Patel S., Arundel C., Deedwania P., Faselis C., Allman R.M., Zhang S., Morgan C.J. (2022). In-Hospital Weight Loss and Outcomes in Patients with Heart Failure. J. Card. Fail..

[8] Naderi N., Chenaghlou M., Mirtajaddini M., Norouzi Z., Mohammadi N., Amin A., Taghavi S., Pasha H., Golpira R. (2022). Predictors of readmission in hospitalized heart failure patients. J. Cardiovasc. Thorac. Res..

[9] Delgado J.F., Ferrero Gregori A., Fernández L.M., Claret R.B., Sepúlveda A.G., Fernández-Avilés F., González-Juanatey J.R., García R.V., Otero M.R., Segovia Cubero J. (2019). Patient-Associated Predictors of 15- and 30-Day Readmission After Hospitalization for Acute Heart Failure. Curr. Heart Fail. Rep..

[10] van Kimmenade R.R.J., Bakker J.A., Crijns H.J.G.M., van Dieijen-Visser M.P., Pinto Y.M. (2004). The value of (NT-pro) BNP in the diagnosis, prognosis and treatment of congestive heart failure. Neth. Heart J..

[11] Díaz-Gómez J.L., Mayo P.H., Koenig S.J. (2021). Point-of-Care Ultrasonography. N. Engl. J. Med..

[12] Pérez-Herrero S., Lorenzo-Villalba N., Urbano E., Sánchez-Sauce B., Aguilar-Rodríguez F., Bernabeu-Wittel M., Garcia-Alonso R., Soler-Rangel L., Trapiello-Valbuena F., Garcia-García A. (2022). Prognostic Significance of Lung and Cava Vein Ultrasound in Elderly Patients Admitted for Acute Heart Failure: PROFUND-IC Registry Analysis. J. Clin. Med..

[13] Gargani L., Girerd N., Platz E., Pellicori P., Stankovic I., Palazzuoli A., Pivetta E., Miglioranza M.H., Soliman-Aboumarie H., Agricola E. (2023). Lung ultrasound in acute and chronic heart failure: A clinical consensus statement of the European Association of Cardiovascular Imaging (EACVI). Eur. Heart J.-Cardiovasc. Imaging.

[14] Sampath-Kumar R., Ben-Yehuda O. (2023). Inferior vena cava diameter and risk of acute decompensated heart failure rehospitalisations. Open Heart.

[15] Wang Y., Shi D., Liu F., Xu P., Ma M. (2021). Prognostic Value of Lung Ultrasound for Clinical Outcomes in Heart Failure Patients: A Systematic Review and Meta-Analysis. Arq. Bras. Cardiol..

[16] McKee P.A., Castelli W.P., McNamara P.M., Kannel W.B. (1971). The Natural History of Congestive Heart Failure: The Framingham Study. N. Engl. J. Med..

[17] Platz E., Merz A.A., Jhund P.S., Vazir A., Campbell R., McMurray J.J. (2017). Dynamic changes and prognostic value of pulmonary congestion by lung ultrasound in acute and chronic heart failure: A systematic review. Eur. J. Heart Fail.

[18] Vizioli L., Ciccarese F., Forti P., Chiesa A.M., Giovagnoli M., Mughetti M., Zompatori M., Zoli M. (2017). Integrated Use of Lung Ultrasound and Chest X-Ray in the Detection of Interstitial Lung Disease. Respiration.

[19] Furtado S., Reis L. (2019). Inferior vena cava evaluation in fluid therapy decision making in intensive care: Practical implications. Rev. Bras. Ter. Intensiv..

[20] Gargani L. (2011). Lung ultrasound: A new tool for the cardiologist. Cardiovasc. Ultrasound.

[21] Mojoli F., Bouhemad B., Mongodi S., Lichtenstein D. (2019). Lung Ultrasound for Critically Ill Patients. Am. J. Respir. Crit. Care Med..

[22] Pang P.S., Russell F.M., Ehrman R., Ferre R., Gargani L., Levy P.D., Noble V., Lane K.A., Li X., Collins S.P. (2021). Lung Ultrasound–Guided Emergency Department Management of Acute Heart Failure (BLUSHED-AHF). JACC Heart Fail..

[23] Burgos L.M., Baro Vila R.C., Ballari F.N., Goyeneche A., Costabel J.P., Muñoz F., Spaccavento A., Fasan M.A., Suárez L.L., Vivas M. (2024). Inferior vena CAVA and lung ultraSound-guided therapy in acute heart failure: A randomized pilot study (CAVAL US-AHF study). Am. Heart J..

[24] Goonewardena S.N., Gemignani A., Ronan A., Vasaiwala S., Blair J., Brennan J.M., Shah D.P., Spencer K.T. (2008). Comparison of Hand-Carried Ultrasound Assessment of the Inferior Vena Cava and N-Terminal Pro-Brain Natriuretic Peptide for Predicting Readmission After Hospitalization for Acute Decompensated Heart Failure. JACC Cardiovasc. Imaging.

[25] Patnaik S., Davila C.D., Lu M., Alhamshari Y., Shah M., Jorde U.P., Pressman G.S., Banerji S. (2020). Clinical correlates of hand-held ultrasound-guided assessments of the inferior vena cava in patients with acute decompensated heart failure. Echocardiography.

[26] Wang Y., Pu X., Zhu Z., Sun W., Xue L., Ye J. (2023). Handgrip strength and the prognosis of patients with heart failure: A meta-analysis. Clin. Cardiol..

[27] Picano E., Scali M.C., Ciampi Q., Lichtenstein D. (2018). Lung Ultrasound for the Cardiologist. JACC Cardiovasc. Imaging.

[28] Choi E.S., Wiseman T., Betihavas V. (2021). Biomedical, Socioeconomic and Demographic Predictors of Heart Failure Readmissions: A Systematic Review. Heart Lung Circ..

[29] McQuade C.N., Mizus M., Wald J.W., Goldberg L., Jessup M., Umscheid C.A. (2017). Brain-Type Natriuretic Peptide and Amino-Terminal Pro–Brain-Type Natriuretic Peptide Discharge Thresholds for Acute Decompensated Heart Failure: A Systematic Review. Ann. Intern. Med..

[30] Formiga F., Chivite D., Corbella X., Conde-Martel A., Arévalo-Lorido J.C., Trullàs J.C., Silvestre J.P., García S.C., Manzano L., Montero-Pérez-Barquero M. (2019). Influence of potassium levels on one-year outcomes in elderly patients with acute heart failure. Eur. J. Intern. Med..

